# A preliminary study of proximal realignment combination surgery in the treatment of adolescent habitual patellar dislocation

**DOI:** 10.1186/s12891-023-06206-2

**Published:** 2023-02-07

**Authors:** Wei Li, De-Bao Zhang, Sheng-Ming Xu, Huang-Yi Bi, Gui-Shan Gu

**Affiliations:** Department of Orthopedics, the First Bethune Hospital of Jilin University, Street Xinmin 71, Changchun, China

**Keywords:** Habitual patellar dislocation, Medial patellofemoral ligament

## Abstract

**Objective:**

A combination of lateral soft tissue release, medial soft tissue contraction, vastus medialis anterior placement, medial patellofemoral ligament reconstruction, and rectus femoris insertion reconstruction are introduced in the treatment of habitual patellar dislocation in adolescents.

**Methods:**

A retrospective analysis was performed on 12 patients (17 knees) with habitual patellar dislocation and unclosed epiphyses who underwent surgical treatment at the First Hospital of Jilin University from May 2017 to November 2021. The Lysholm scores and Q angle were collected preoperatively and at final follow-up and were compared.

**Results:**

Twelve patients (4 boys and 8 girls) aged 10–15 years were retrospectively analysed, who followed up for an average of 21 months (5–48 months). The range of motion of the knee joint returned to normal in all patients, and no cases of complications including surgical site infection, joint stiffness, or patellar re-dislocation occurred. The mean Lysholm scores and Q angles improved from 73.9, and 19.6° preoperatively to 91.7, and 13.9° at the final follow-up, respectively.

**Conclusion:**

The preliminary effect of the combination surgery for habitual patellar dislocation in adolescents was satisfactory.

## Introduction

Habitual patellar dislocation(also called obligatory patellar dislocation) is characterized by the gradual lateral displacement of the patella that progresses to dislocation during knee flexion and spontaneous repositioning after knee straightening. It is known that dislocation of the patella can cause patellofemoral pain and degenerative arthritis [1]. The disorder occurs with cyclic knee flexion and extension, and its main pathological factors are contracture of the lateral soft tissue of the knee joint, hypoplasia of the femoral trochlea, increased Q angle, increased tibial tubercle-trochlear groove (TT-TG) distance, and patella alta. Some scholars have pointed out that the vastus medialis obliquus is the most important factor in maintaining the stability of the medial patella [2–4]. Currently, surgery is the recommended [5–7] treatment for this condition;however, because of the complexity of its pathology, a multitude of surgical procedures have been designed.Joo et al. published a four in one technique consisting of lateral retinacular release, semitendinosus tenodesis, Roux Goldthwait partial patellar tendon transposition and proximal tube realignment of the patella in patients with habitual patellar dislocation [8, 9], but complications including surgical site infections, knee stiffness, and recurrent dislocation may occur after surgical repair of habitual patellar dislocation in adolescents.

**Fig. 1:**
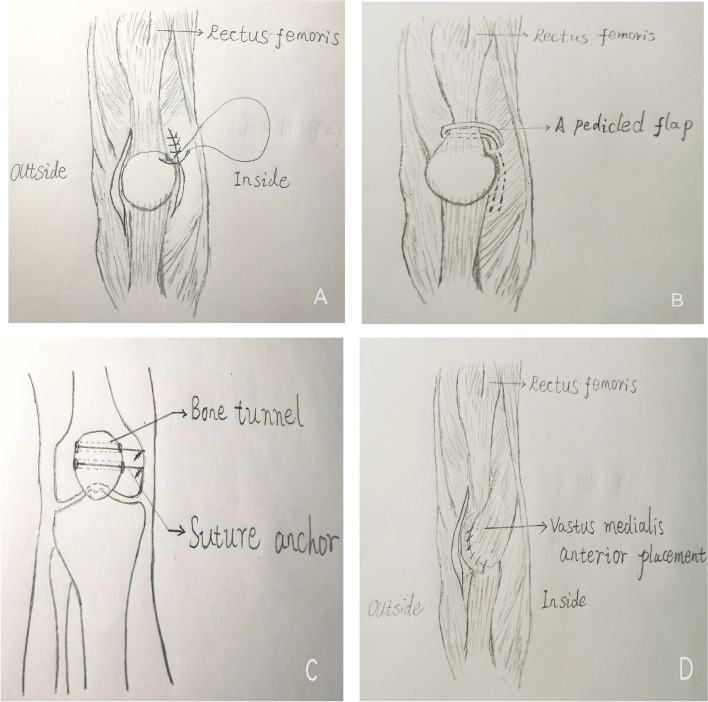
**A** Lateral soft tissue release, medial soft tissue contraction. **B** Rectus femoris insertion reconstructions. **C** Medial patellofemoral ligament reconstruction.  **D** Vastus medialis anterior placement

As no single procedure has been deemed effective in fully treating this condition and combining different procedures becomes necessary, we planned to study the effectiveness of combining five kinds of reconstruction in a single procedure. In this retrospective study, lateral soft tissue release, medial soft tissue contraction, vastus medialis anterior placement, medial patellofemoral ligament reconstruction, and rectus femoris insertion reconstructions were performed in adolescent patients for the treatment of habitual patellar dislocation(Fig. 1) A-D), and the functional and radiographic outcomes were also reported.

## Materials and methods

We performed a retrospective analysis of the clinical data of 12 patients (4 boys, 8 girls; 17 knees) with habitual patellar dislocation and unclosed epiphysis who underwent treatment at the First Hospital of Jilin University from May 2017 to November 2021. The patients were aged between 10 and 15 years. The main symptoms of the patients were knee flexion, gradual patella prolapse with the increasing knee flexion angle, complete patella prolapse at a larger flexion angle, and nearly complete dislocation reduction with knee extension. Inclusion criteria: (1) habitual patellar dislocation; (2) an unclosed epiphysis or one that was in a state prior to or at the peak of growth; (3) no prior fracture of the affected limb in the past,ligament reconstruction or knee joint-related surgery; and (4) no congenital multiple ligament laxity. Patients with genu torsional deformities of the tibia and femur were excluded.

Patients with congenital patellar dislocation, recurrent patellar dislocation, patellar dislocation caused by neuromyopathy, no obvious atrophy, or poor muscle strength of the vastus medialis were excluded from the analysis. The preoperative Lysholm scores and Q angles were collected and compared with the corresponding values at the final follow-up. To measure the Q angle, the patient laid supine on the examination bed, and the lower limb muscles were relaxed and in the neutral position so that the patella was in the anatomical position, and the second toe of the affected side was kept upwards, perpendicular to the examination bed. The body surface method was used to locate the midpoint of the patella while the skin was held in place. Then the anterior superior iliac spine and the tibial tubercle were located, and a green laser pen was used to position the quadriceps force line (the line from the anterior superior iliac spine to the midpoint of the patella) and the patellar ligament force line (the line from the midpoint of the patella to the tibial tubercle). The sharp angle formed by the above two lines was measured by a protractor and recorded as the Q angle in the straight position. Our study was approved by the ethical committee of the First Bethune Hospital of Jilin University.

## Surgical procedure

All patients underwent surgery under general anaesthesia without a pneumatic tourniquet. After anaesthesia had been induced, the limitations of knee motion, the angle of patellar dislocation, and the symmetry of the bilateral vastus medialis muscle were assessed.

**Fig. 2:**
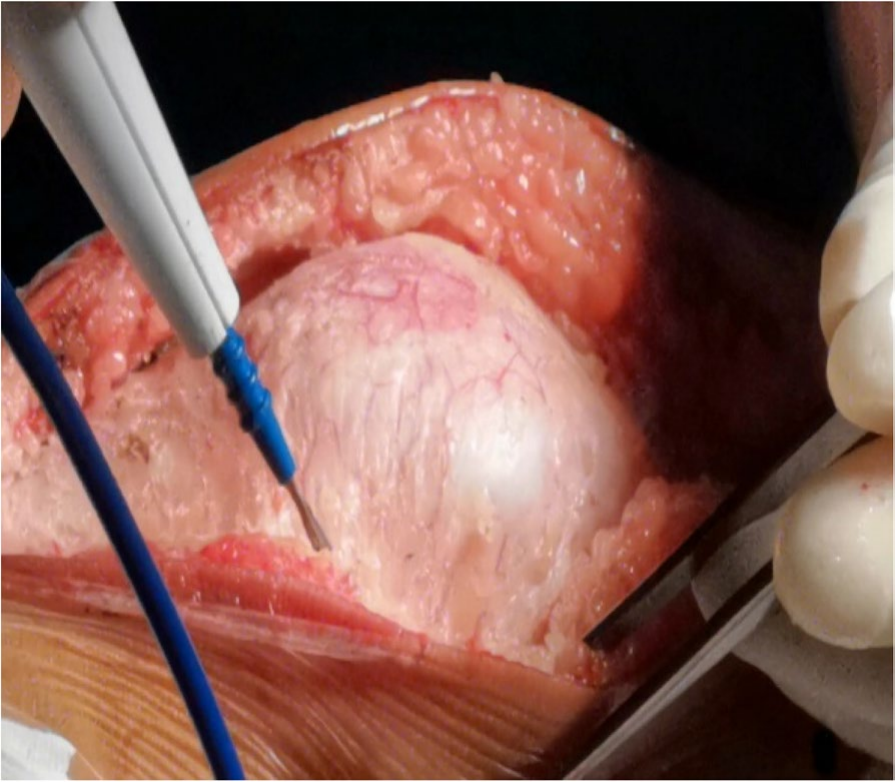
Midline incision of the knee joint; the fascia was exposed on both sides; electrocoagulation was used to stop bleeding

**Fig. 3:**
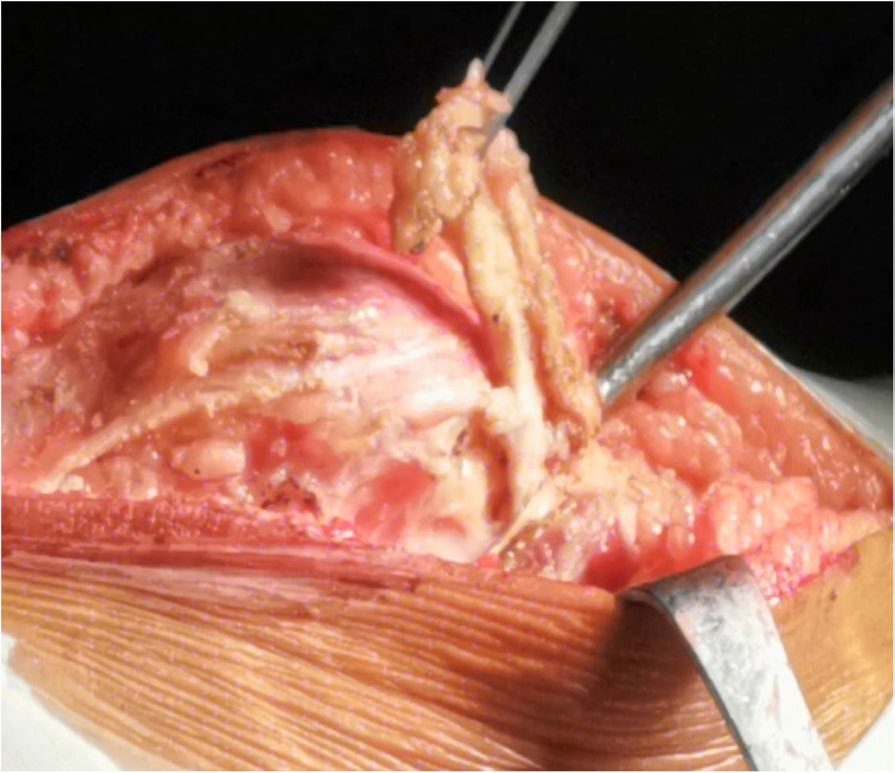
A pedicled flap was created and extended from 0.5 cm medial to the superior pole of the patella to the distal end of the patella, and the articular capsule medial to the knee joint is opened

**Fig. 4:**
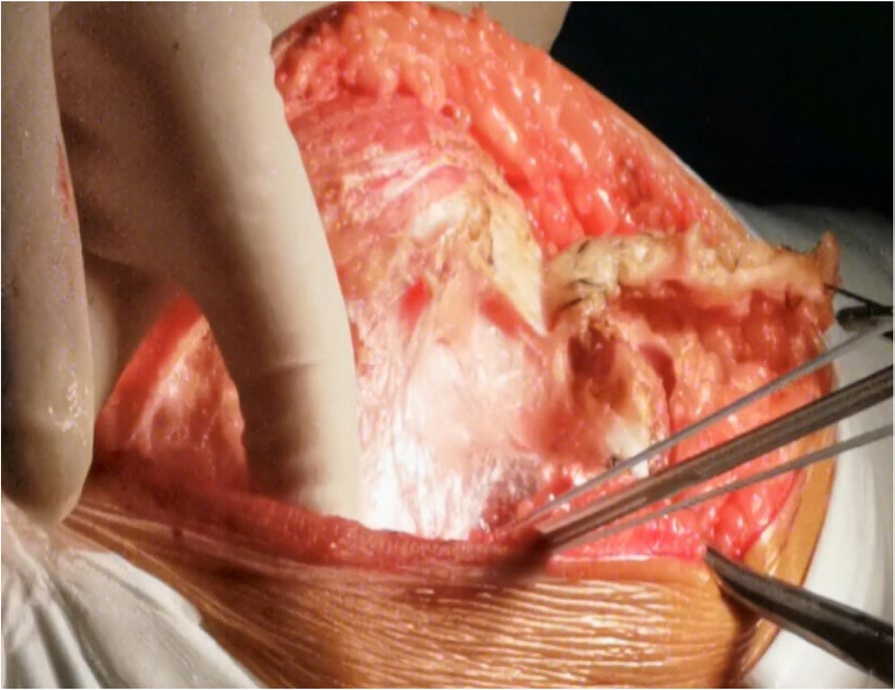
A suture anchor is placed in the proximal femur

**Fig. 5:**
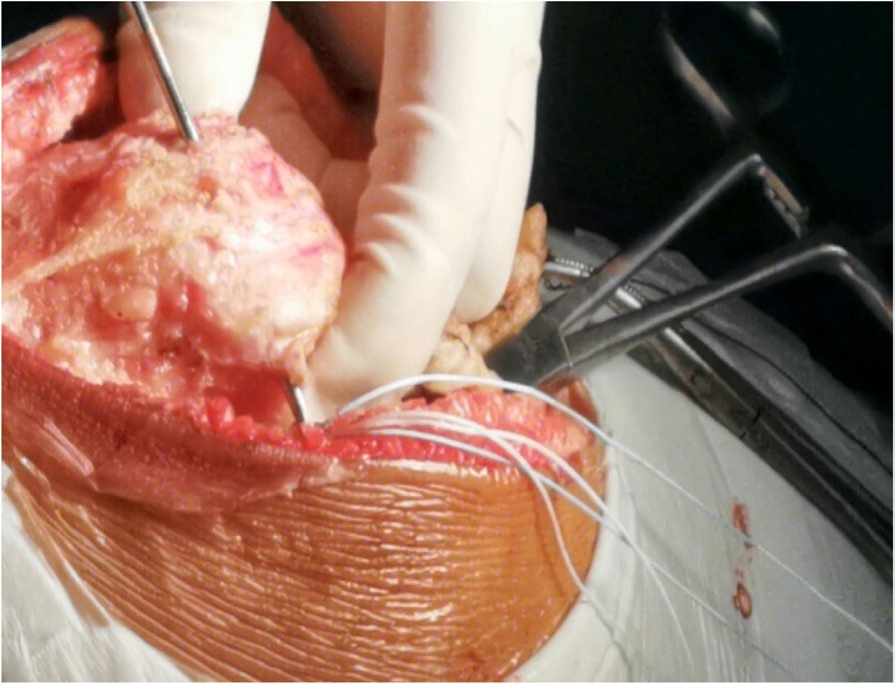
2.0 mm Kirschner wire is used to build a tunnel through the middle of the patella

**Fig. 6:**
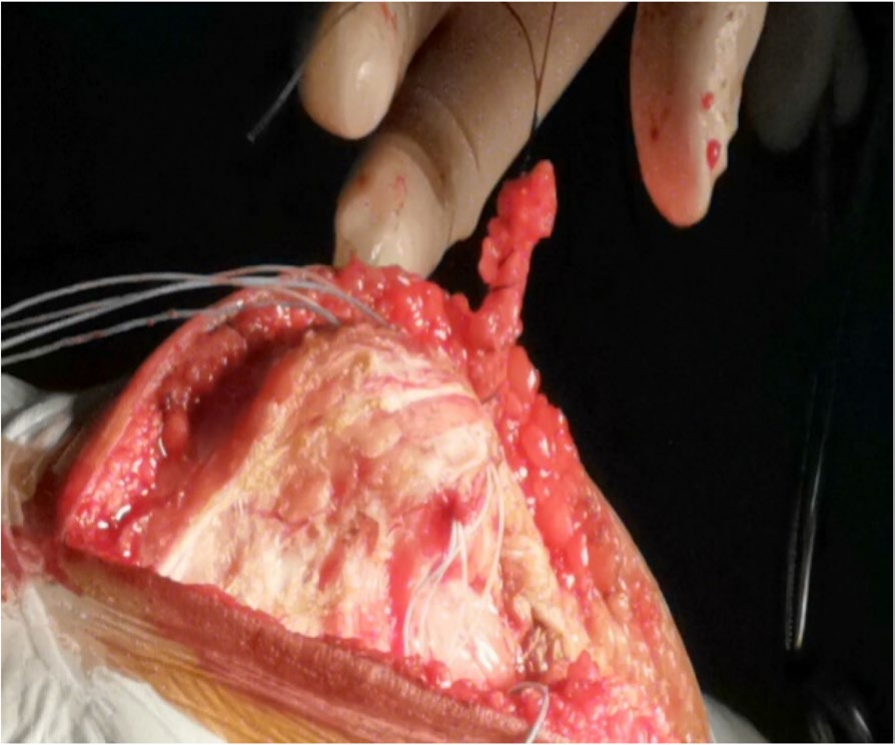
Four wires of the suture anchor are passed through the two bone tunnels

**Fig. 7:**
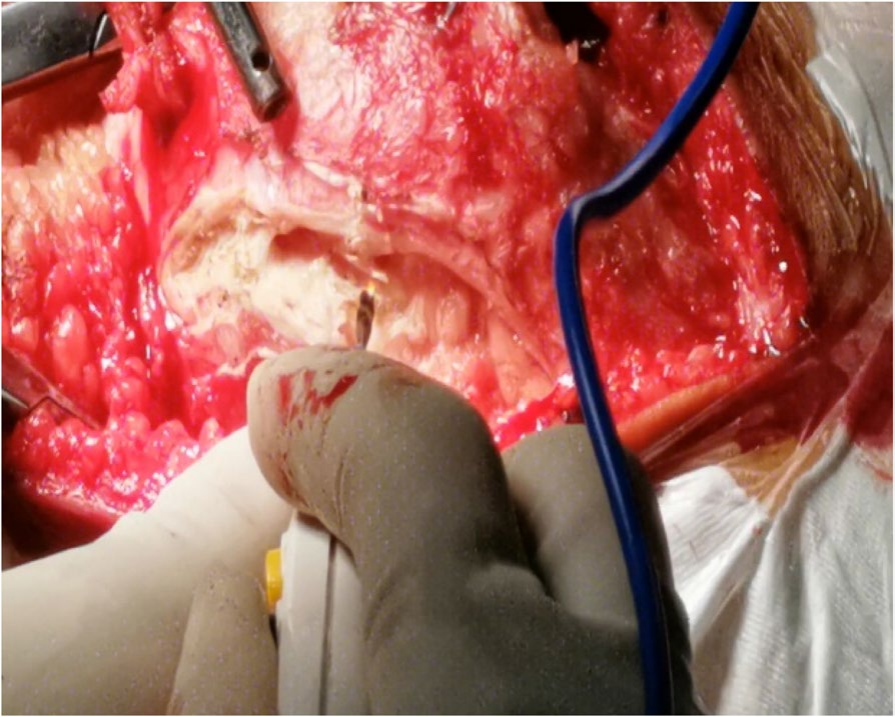
Image showing complete release of the lateral soft tissue

A 10-cm midline incision was made over the knee joint and subsequently, the patella and patellar ligament were exposed (Fig. 2) through the skin incision and superficial fascia. A 0.5-cm wide, 6-cm long pedicled flap was created from 0.5 cm medial to the superior pole of the patella, extended to the distal end of the patella (Fig. 3) and strengthened by threads. The flap was fully disassociated at the point of insertion of the vastus medialis. The suture anchor was placed 1 cm above the medial femoral condyle (Fig. 4), followed by the application of 2.0 mm Kirschner wires to establish a bone tunnel from the lateral mid-superior quarter to the middle of the patella towards the medial patellar edge (Fig. 5). Another suture anchor was placed 1 cm above the articular surface of the medial femoral condyle and anterior to the posterior femoral cortex, and the four wires of this suture anchor were passed through the two bone tunnels (Fig. 6). The soft tissue lateral to the patella was released, while the reduction of the patella was examined until the patella was completely in position during knee flexion (Fig. 7). At this point, the medial and lateral soft tissues of the patella were completely released.

**Fig. 8:**
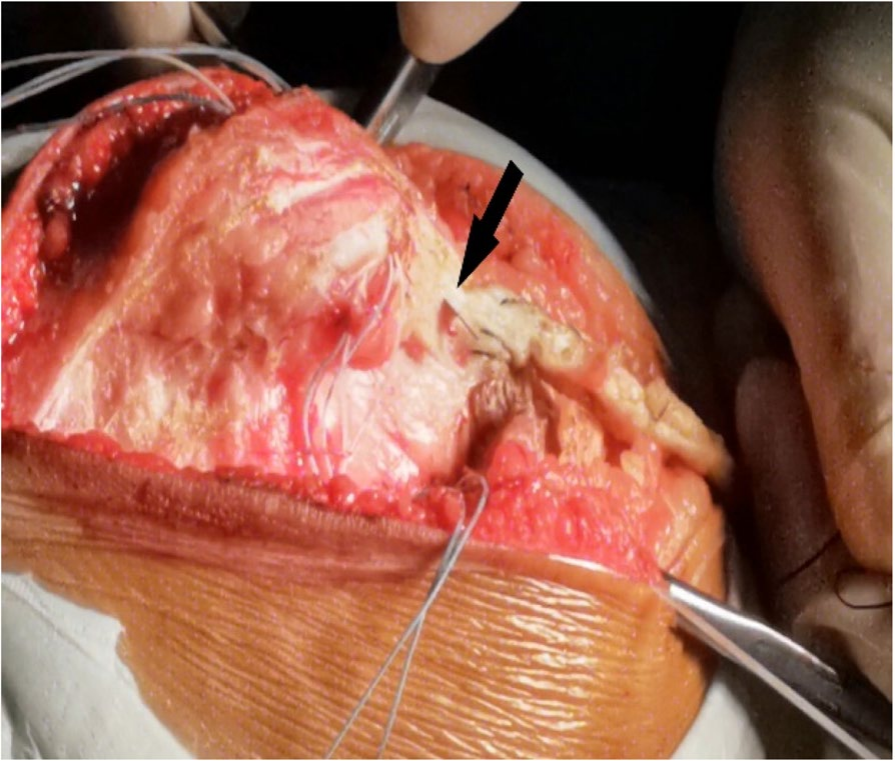
A scalpel (black arrow) is used to establish a tendinous tunnel through rectus femoris

**Fig. 9:**
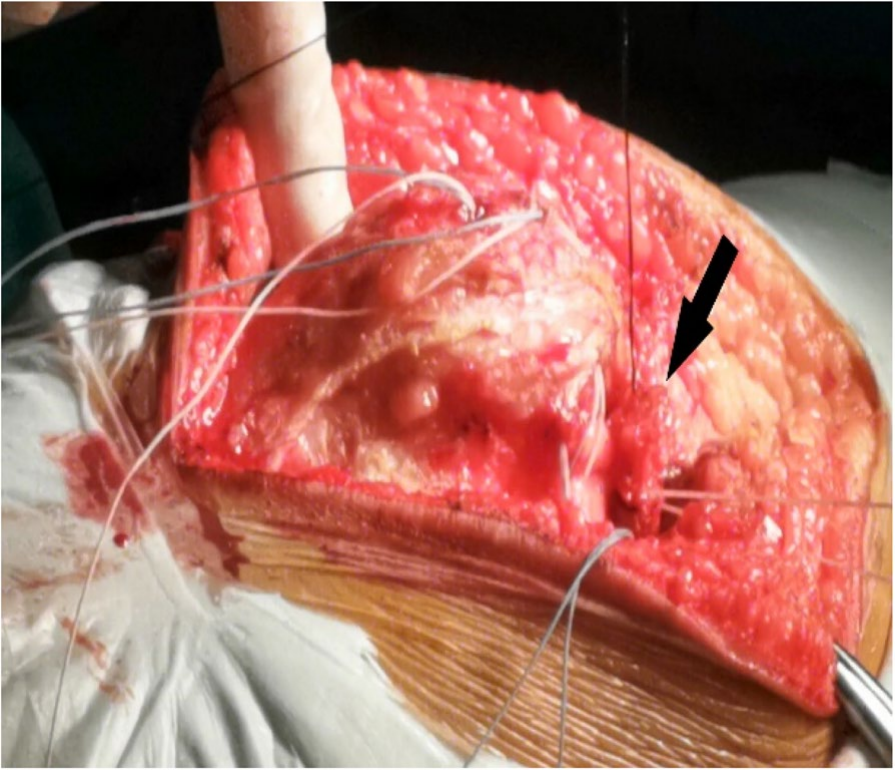
The black arrow shows a folded pedicled flap

A tendinous tunnel measuring 0.5-cm wide was established by penetrating into the rectus femoris tendon 0.5 cm from the upper pole of the patella with a sharp knife blade (Fig. 8). Thereafter, the tendinous tunnel of the rectus femoris muscle was penetrated outwards, the strengthened pedicled flap was turned over to the medial side, and the rectus femoris tendon was pulled medially by the flap, while the patella was pushed medially to the reduced state. The pedicled flap was strengthened by the wires of the suture anchor and sutured to the distal end of the rectus femoris tendon after fixing it with surgical knots to the wires of the first suture anchor (Fig. 9). These steps of completed the medial transfer of the rectus femoris.

**Fig. 10:**
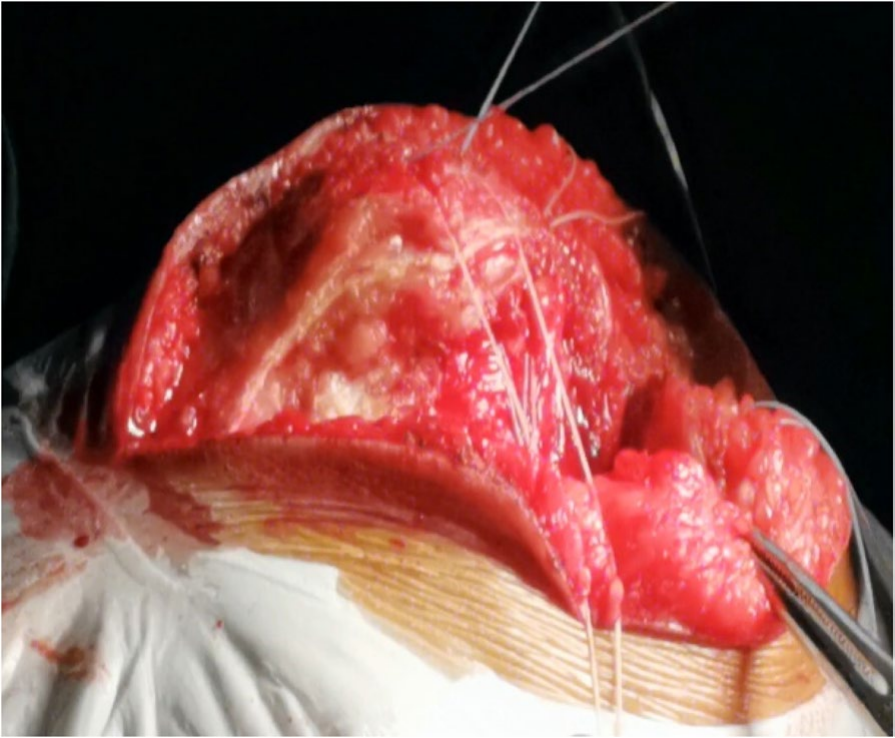
The knee is flexed at 60° and fixation is performed with knotted threads

Next, we performed reconstruction of the patellofemoral ligament by first flexing the knee joint at 60°, tightening four wires of the second suture anchor and fixing them with cross-knots for reduction of the patella (Fig. 10). Then, we rechecked the positioning of the patella in the patellar trochlea for the knee flexion and extension positions, which indicated that complete reduction was obtained.

**Fig. 11:**
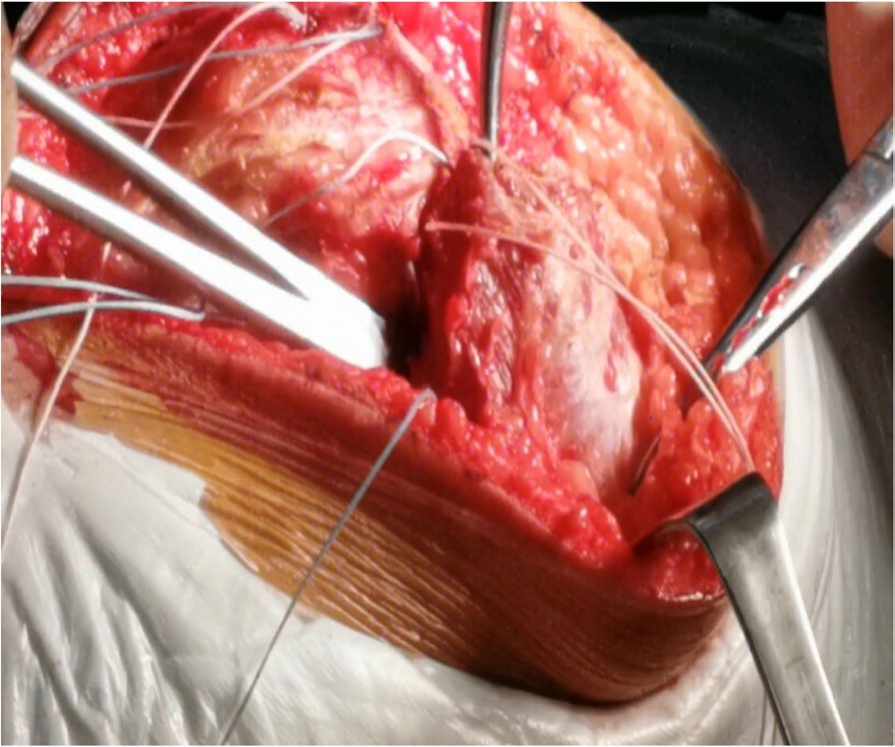
Reconstruction of the vastus medialis (black arrow) insertion

Next, we performed reconstruction of the vastus medialis muscle insertion by pulling it inwards and downwards; then, the muscle was attached to the medial patella and firmly fixed with the wires of the first suture anchor (Fig. 11).

Finally, the lateral patellar soft tissue was sutured and the joint capsule at the medial edge of the patella was contracted, followed by strengthening of the soft tissue and suturing it to the medial patellar fascia and periosteum.

**Fig. 12:**
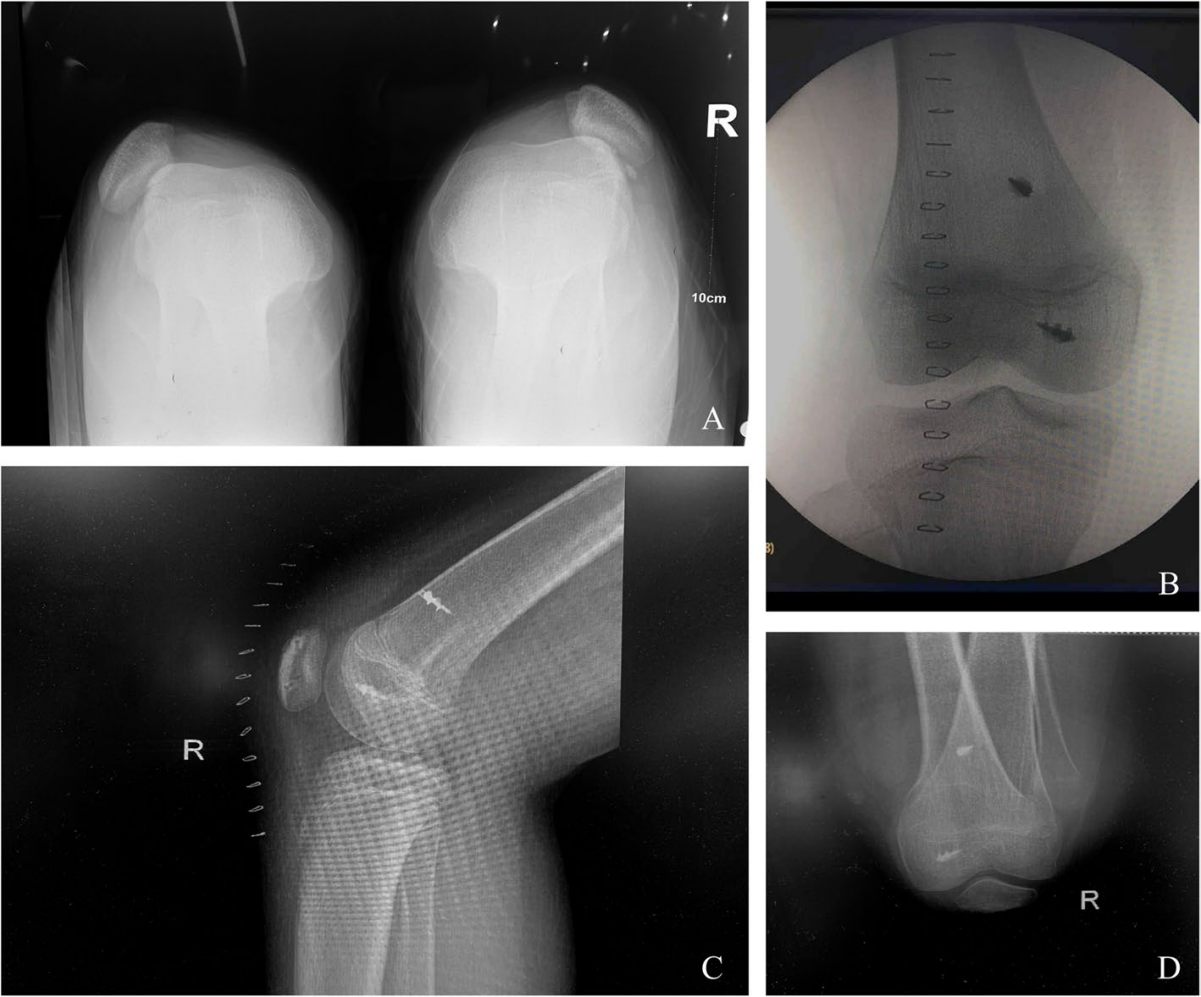
A–D. **A**: Preoperative computed tomograms of one patient. Postoperative anteroposterior (**B**), lateral (**C**), and axial (**D**) X-ray views of the knee joint

Preoperative computed tomograms of one patient are shown in Fig. 12 A–D.

**Fig. 13:**
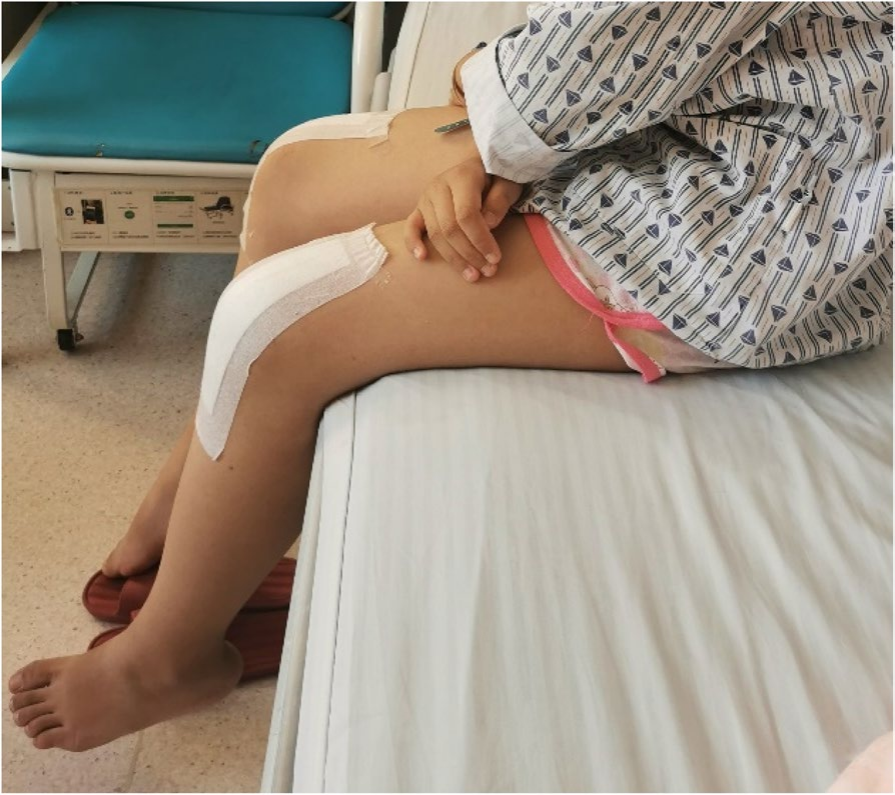
Picture showing that flexion of the bilateral knee joint was 90°14 days after bilateral patellar habitual dislocation

Drainage tubes were placed in all patients after the incision and removed 48 h later. After the operation, the knee joint of the affected side was fixed at 20°with external fixation brace. Having been taught to perform isometric and isotonic contractions of the quadriceps femoris before the operation, all patients were asked to perform this exercise again after the anesthesia had wore off. Forty-eight hours after the drainage tube was removed, the knee joint was transferred from passive flexion function exercise, so long as the patient could tolerate the pain and reached 90°knee flexion in 2 weeks (Fig. 13),and walking with partial load on crutches, Weight bearing was limited to what the patient could bear bud did not exceed more than 50% of the body weight. The external fixation braces were removed after 6 weeks; within 4 weeks, the range of flexion and extension of the knee joint gradually increased and reached the normal range of motion.

## Statistical analysis

The follow-up data were analysed by SPSS software, and the paired sample t test was performed to compare groups of data. The differences in the clinical variables between the preoperative and postoperative values were considered statistically significant at *P* < 0.05.

## Results

We analysed 12 patients (4 boys, and 8 girls) aged 10–15 years, with an average operation time of 70 min and a mean length of hospital stay of 7 days (Tables 1, and  2). The average Lysholm score increased from 73.9 before the operation to 91.7 after the operation, t =  − 16.629, and the average preoperative and postoperative Q angles were 19.6° and 13.9° (t = 16.494), respectively, showing a marked decrease. Complications including surgical site infection and joint stiffness were not reported for this cohort. The differences were found to be significant(*P*-value < 0.001).

**Table 1:** Baseline data of all 12 patients

Patient. no	Age (years)	Sex	Side	Follow-up (months)
1	13	F	L	23
2	13	F	L	25
3	14	M	R	26
4	10	F	L	12
4	10	F	R	18
5	14	F	L	48
6	14	M	R	17
7	11	M	R	24
8	10	M	R	25
9	12	F	L	26
			R	9
10	15	F	Both	9
11	11	F	Both	27
12	12	F	Both	5

**Table 2:** Preoperative and postoperative Lysholm score and Q angles of all 12 patients

Patient no	Side	Lysholm score		Q angle	
		Preoperative	Postoperative	Preoperative	Postoperative
1		81	90	20°	14°
2		69	87	19°	14°
3		77	95	20°	15°
4	L	70	96	19°	13°
	R	70	96	21°	12°
5		81	94	21°	14°
6		71	91	18°	13°
7		75	95	19°	14°
8		70	92	21°	13°
9	L	75	95	19°	15°
	R	75	91	21°	16°
10	L	75	92	19°	13°
	R	74	91	18°	14°
11	L	75	91	20°	13°
	R	74	90	19°	14°
12	L	70	87	20°	13°
	R	74	86	19°	15°

## Discussion

Habitual patellar dislocation is prevalent among children and adolescents. Some scholar reported that habitual patellar dislocation was a kind of congenital patellar dislocation [10]. In a group of 35 children with patellar dislocation, Gao [11] reported that 23 had habitual patellar dislocation. As the pathological basis of this condition is complex, including the anatomical features, various surgical techniques have been designed for its treatment. In 1964, Gunn [12] proposed that a contracture of the quadriceps femoris can cause patellar dislocation; hence, Joo [9] suggested the use of quadriceps femoris tenolysis for the treatment of patellar dislocation.

The surgical techniques for early habitual patellar dislocation in children can be classified into 3 types: simple bone surgery; muscle and tendon surgery; and joint capsule and ligament surgery. Many surgical procedures include: distal procedures involving the tibial tuberosity,correction of shortness of the quadriceps, MPFL reconstruction and overlapping medial retinaculum suture and Groove-deepening trochleoplasty [13]. However, the simple application of these techniques to any of the operation types can lead to different complications. Patellar ligament transposition reduces the strength of patellar tendon and has a poor effect on patients with obvious patellar displacement and lesions in the intercondylar fossa of the femur [14]. Some patients showed signs of patellofemoral joint degeneration after femoral pulley plasty, and the anterior knee pain was aggravated [15]. In recent years, many scholars have advocated the combined application of multiple surgical methods. Musielak [16] held that to obtain permanent repositioning and good knee function in habitual patellar dislocation, a combination of proximal (patellar) and distal (tibial) reconstruction was needed and used a modifed Grammont and Langenskiöld technique to restore the correct anatomical conditions. Although the causes of habitual patellar dislocation include femoral trochlear dysplasia and a high patella, Mittal did not find this in his research [17], he performed a multiple incomplete transverse incision along the vastus lateralis under the surface of the rectus femoris, iliotibial band and thick antrelateral part of the femur periosteum instead of reconstruction of the medial patellofemoral ligament. Hire [18] considered that lateral retinacular releases and lengthening, Roux Goldthwait patellar tendon hemi-transfer, modified Insall’s proximal “tube” realignment, and quadriceps slide-lengthening to be good oprations for fixed and habitual patella dislocations.

While the main goal of the surgical treatment of habitual patellar dislocation is to correct the aetiology and anatomical abnormality of the patellar dislocation and restore a normal range of motion, the combination approach adopted in this study was based on the release of the lateral soft tissue of knee joint and contraction of the medial joint capsule integrated with vastus medialis anterior placement, medial patellofemoral ligament reconstruction, and rectus femoris insertion reconstruction. For patients with habitual patellar dislocation in adolescence, more soft group procedures are advocated because their epiphyses are not yet closed, whereas single soft group procedures have poor results, so more combined procedures are advocated [19, 20]. This combination of these five reconstructive surgeries covers three aspects of the patella —muscle, bone, and ligament, and achieves satisfactory clinical effects.

The medial patellofemoral ligament is vital for maintaining the static stability of the patella. Matsushita T et al. [21] believed that the main problem in habitual patellar dislocation was disorder of the medial patellofemoral ligament and that reconstruction can solve > 50% of the static stability problems in this disorder.

For the reconstruction of the medial patellofemoral ligament, the semitendinosus tendon, gracilis tendon or patellar ligament, hamstring tendon, allogeneic ligament, and an artificial ligament must be considered. Satisfactory effects in restoring a normal range of motion can be achieved with consideration of all these anatomical structures. For performing surgery in adolescents with habitual patellar dislocation, more attention is necessary to prevent injury and instability of the tissues around the knee joint, which could occur because of the application of autologous tendons.

MPFL double bundle anatomic reconstruction is more consistent with the native MPFL fan-shaped structure in terms of anatomic features [22]. Wang et al. [23] found that both single and double bundle MPFL reconstructions restored patellar stability through their study of adult knee specimens.In this study, double-row fixation with a suture anchor was applied without lapse and no other damage to the tissues of the knee joint occurred. This procedure was also less expensive than the use of an artificial ligament. When considering the fixed position of the reconstructed ligament, the best fit position of the patellar graft is the proximal end of the patellar footprint because its isotonic property has been reported to be favourable [24, 25]. From previous studies, the insertion of the medial patellofemoral ligament is known to cover the proximal two-thirds of the patella in 56.9% of cases, and the insertion was located in the proximal and distal halves of the patella in 41.2% and 1.3% of cases, respectively, and remained attached to the entire medial edge of the patella in another 1.3% of cases [26]. Therefore, the upper-middle quarter of the patella and the med-patellar region were selected to establish the patellar tunnel. We used a 2.0 mm Kirschner wire to establish a patella double channel and a suture anchor was used to reconstruct the medial patella ligament. Applying both these instruments not only ensured better stress distribution but also restored the anatomical and physiological structure of the medial patellofemoral ligament to the maximum extent.

Patellofemoral joint stability is determined by both, the bone structure and soft tissue. Patellofemoral instability is more common in patients with patellar dislocations [27]. Specifically, the static structure consists of articular cartilage on the patellofemoral joint surface and the surrounding supportive tissue, while the dynamic stability structure includes the quadriceps muscle. The vastus medialis muscle, however, is the only dynamic structure that can pull the patella inwards and backwards [28]. In this study, the most important step for vastus medialis insertion reconstruction surgery was physical examination of the patient before surgery. If the vastus medialis muscle is severely atrophied, it should not be treated surgically.

Since the epiphysis of adolescent patients with habitual patellar dislocation has not been completely closed, soft tissue surgery is important, with the ultimate goal of improving the Q angle. Abnormal changes in the Q angle may create an unequal distribution of pressure across the patellar cartilaginous surface and may present with associated symptoms of patellofemoral disease [29]. Wang et al. [30] found that, in clinical conditions such as female patients, the Q angle was not significantly increased in the extended position, but the Q angle was significantly changed in the flexed knee position compared with the extended position at 30°, to consider the possibility of recurrent patellar dislocation.If an abnormal Q-angle can be improved by surgery (meaning that the angle can be corrected to < 15°), the proximal force line of the patella can be improved, thereby treating the patella dislocation. Enlargement of Q angle is the cause of pain and operation failure [31, 32].

Distal reconstruction is required for the improvement of Q angle, but there wsa no distal reconstruction in this combined operation. However, during the follow-up of more than one year, we found that the Q angle was improved in varying degrees. The reasons are as follows: all the patients treated are teenagers whose epiphysis has not been closed. After surgical treatment, with the obvious improvement of the patients' prepatellar pain, their walking gait tends to be normal, and the stress of the lower limbs also changes. With the increase of age, epiphyseal development tends to close gradually, improving the lower limb force line. This study is consistent with the results reported by Li Zhibin [33], who used arthroscopic-assisted medial patellofemoral ligament reconstruction to treat recurrent patellar dislocation. 88 patients were followed up 3 months after operation, the Q angle improved significantly. Therefore, the author is carrying out a multicenter study on the changes of gait and lower limb stress during walking in adolescents with habitual patellar dislocation after combined surgery, in order to further clarify the related reasons for the improvement of Q angle.We were further able to maintain the patellar range of motion in the femoral trochlea when the knee was flexed > 20° by pulling the rectus femoris tendon inwards using a pedicled fascia flap, which has not been reported thus far.

None of the patients in this study had an obvious bayonet sign during the preoperative physical examination.The drawer test, Lachman test and axis shift test were negative in all patients,and no patients had a history of knee joint trauma before the operation. Combined intra- and extra-articular reconstruction may provide a more normal restoration of knee kinematics after anterior cruciate ligament injury with concomitant anterolateral rotational laxity, and extra-articular tenodesis is necessary to restore intact kinematics when a lateral capsule lesion is present [34]. Fortunately, there were no problems with knee rotation stability in any patient in this study.

The limitations of this study are as follows: (1) This was a retrospective study, and the sample size was relatively small; (2) The patient interview time was relatively short, and there was lack of long-term follow-up results; the findings reported here thus reflect only the short-term clinical treatment effect. In future work, we will continue to follow up the medical records of the patients of this study. In the diagnosis and treatment of adolescent patellar dislocation, we should pay more attention to preoperative risk factor assessment, increase the sample size, and continue to evaluate the efficacy of this procedure.

## Conclusion

In conclusion, the combined surgery for habitual patellar dislocation in younger adolescents has good functional outcomes, does not require an autologous or artificial tendon, and is less expensive than other similar surgeries. Long-term follow-up with a larger sample size is required to remedy the limitations of this study.

## Data Availability

The datasets used and/or analysed during the current study are available from the corresponding author on reasonable request.
